# Value of Abdominal Belt after Cœliotomies

**Published:** 1896-08

**Authors:** Stuart McGuire

**Affiliations:** Richmond, Va., Professor of Principes of Surgery, Universivy College of Medicine


For the Texas Medical Journal.

### ON THE PROPHYLACTIC VALUE OF AN ABDOMINAL BELT AFTER CŒLIOTOMIES.

BY STUART M'GUIRE, M. D., OF RICHMOND, VA., PROFESSOR OF PRINCIPES OF SURGERY, UNIVERSIVY COLLEGE OF MEDICINE.

[Read before the Richmond Academy of Medicine and Surgery, April 14, 1896.]

DURING the last five years, I have had charge of the after-treatment of nearly three hundred cases of abdominal section. On leaving bed, each case has been fitted with an abdominal belt, and instructed to wear it for one year; and, though ventral hernia has been rare, I have become convinced that the integrity of the abdominal walls was due rather to accurate suturing and to prolonged confinement in a recumbent posture than to any virtue of the artificial support. This opinion was so at variance with my preconceived ideas on the subject that I determined to write to some of the leading surgeons and gynecologists of the country to ascertain their practice. The following is an abstract of answers received:

Dr. John Ashhurst, Jr., of Philadelphia, said that he did not invariably use an abdominal belt after cœliotomy, but that he thought upon the whole it was a useful precaution to employ.

Dr. W. T. Bull, of New York, said he employed an abdominal belt after abdominal sections for at least a year, when the wound healed by primary union, undefinitely if it healed by granulation. He was convinced of its value in preventing hernia, although the condition might develop in spite of its use, that it was the only safeguard we had, and failure to use it might, in large wounds, lead to voluminous and disabling protrusions.

Dr. David W. Cheever, of Boston, said he used an abdominal belt with a pad, over the incision, after suprapubic operations, and that he saw no reason to doubt its value as a prophylactic against hernia.

Dr. P. S. Connor, of Cincinnati, said he used an abdominal belt, but he thought it of very doubtful value.

Dr. W. H. Carmalt, of New Haven, said he used an abdominal belt after laparotomies, and that he would continue to do so until some reliable surgeon or gynecologist told him that he had dispensed with it, and found it unnecessary.

Dr. John B. Deaver, of Philadelphia, said he used an abdominal belt, and was convinced of its value.

Dr. W. E. B. Davis, of Birmingham, said he used an abdominal belt in deference to the practice of others, but that he had no positive conviction of its value.

Dr. A. G. Gerster, of New York, said that he used the abdominal belt only where the wound healed by granulation, and that he thought it of conditional value in retarding hernia.

Dr. J. McFadden Gaston, of Atlanta, said he used an abdominal belt with a large compress after all sections for abdominal tumors to restore pressure upon the viscera, and that it could not be dispensed with for a month after such operations without incurring risk of hernia.

Dr. J. B. S. Holmes, of Atlanta, said he had abandoned the use of the abdominal belt, and that he had never realized any benefit from its employment.

Dr. Wm. S. Halstead, of Baltimore, said he never used an abdominal belt, and did not believe it was of any value.

Dr. Howard A. Kelley, of Baltimore, said he used an abdominal belt only in exceptional cases, and then for comfort; it was of no value as a preventive of hernia.

Dr. W. W. Keen, of Philadelphia, said he used an abdominal belt after all cœliotomies, and was convinced of its value.

Dr. L. C. Lane, of San Francisco, said he used an abdominal belt, and was confident it retarded the development of hernia.

Dr. Claudius H. Mastin, of Mobile, said he did not use the abdominal belt, nor did he think it possible that it could prevent hernia, that he closed the wound with three layers of buried cat gut sutures, sealing it with iodoform collodion, and never took the dressing off, unless indications arose, until it was perfectly healed; that he had had but one case of hernia in his practice, and that occurred where a glass drainage tube had been inserted, and that he believed, in this case, the accident could have been prevented by care.

Dr. Chas. McBurney, of New York, said he generally advised the use of a well-fitting abdominal belt after an abdominal section, and that if the belt fitted perfectly every part of the ab domen, he thought it useful; otherwise, in making uneven support, it did positive harm.

Dr. H. H. Mudd, of St. Louis, said that he very rarely used an abdominal belt, and that he did not believe it was of any value in preventing the occurrence of hernia.

Dr. J. Ewing Mears, of Philadelphia, said he advised the use of an abdominal belt for a period of from three to six months after a section, and that he was convinced of its value.

Dr. Thos. G. Morton, of Philadelphia, said he occasionally used an abdominal belt, especially in the obese, and that it seemed to be of value in some unusual cases.

Dr. E. M. Moore, of Rochester, said he never used an abdominal belt, and he thought it had no value.

Dr. J. B. Murphy, of Chicago, said he did not use an abdominal belt after any cœliotomy, as he was convinced it was of no value in preventing hernia.

Dr. Henry O. Marcy, of Boston, said he had not for the past ten years used an abdominal belt after cœliotomy; that in three hundred cases where he had closed the wound with tendon sutures and sealed with collodion, he had but two hernias,—one from suppuration, and one from general pouching of aponeurosis between the separated recti.

Dr. Chas. B. Nancrede, of Ann Arbor, said that he used an abdominal belt after all cœliotomies, as he believed it prevented hernia by lessing the effect of sudden or violent efforts.

Dr. T. F. Prewitt, of St. Louis, said he used an abdominal belt after cœliotomies, and he thought it of service.

Dr. L. S. Pilcher, of Brooklyn, said he advised the use of an abdominal belt for from six to nine months after an operation, and he was convinced of its value.

Dr. Joseph Price, of Philadelphia, said he never used an abdominal belt, as he was convinced it was of no use in preventing hernia.

Dr. John H. Packard, of Philadelphia, said he did not use an abdominal belt after removal of the primary dressings, except in some cases for its moral effect, and that he believed it was of value only when the wound was very extensive, or when the walls were very lax.

Dr. Roswell Park, of Buffalo, said he very rarely used an abdominal belt, and that it was only of value in very fat patients.

Dr. John B. Roberts, of Philadelphia, said he usually, but not always, employed an abdominal belt; that he had no absolute assurance of its value, but liked it in cases of pendulous abdomen.

Dr. L. A. Stimson, of New York, said he rarely used an abdominal belt after cœliotomies, but was convinced it was of value in some cases.

Dr. N. Senn, of Chicago, said he invariably directed the use of a proper abdominal support after every abdominal section, and insisted on the patient wearing it from six months to a year, as he believed the bandage useful in preventing ventral hernia.

Dr. W. S. Tremaine, of Buffalo, said he used a belt after cœliotomies, and was convinced of its value.

Dr. L. M. Tiffany, of Baltimore, said he used a belt after cœliotomies, and was convinced of its value.

Dr. A. Vander Veer, of Albany, said he used a belt after cœliotomies for comfort, but did not think it tended to prevent the formation of hernia.

Dr. R. O. Weir, of New York, said he did not use a belt when the wound had been closed by suturing and healed by primary union, but usually employed it when the wound had not been sutured and healed by granulation. As a preventive of hernia, he thought it of doubtful value, but useful as a placebo.

Dr. J. E. Warren, of Boston, said he used the belt after cœliotomies, and he thought it had some influence in preventing distension of the cicatrix after unusual exercise.

It will be seen that the majority of the writers employ an abdominal belt after cœliotomies,—some from conviction, some from doubt, and some from indifference. The fact, however, that a single competent observer has discarded its use, and found no reason to regret abandoning artificial support, proves that in the large majority of cases it is unnecessary. Because an abdominal belt is indicated in some instances, is no reason why it should be employed in all cases. Routine practice is bad practice.

I protest against the use of the belt, not only because of expense, which to some is of considerable importance, but also because of the irritation, annoyance, etc., and I believe it will follow the obstetrical binder, being unnecessary.

DISCUSSION.

Dr. Aaron Jeffrey had, for a number of years, discarded the obstetric binder, but in the more recent years had returned to its use for the comfort of the patient, if nothing more. He agrees with Dr. McGuire concerning the use of the abdominal belt after laparotomies.

Dr. Wm. S. Gordon remarked that sometimes syncope results from the shock of suddenly relieved pressure, as in dropsy. Where the abdominal walls are released, a binder should be used, but properly applied. Mechanical stimulus is a powerful excitant of muscular tissue, keeping up its tone. For this purpose the binder should be employed.

Dr. Ed. McGuire said the application of support after laparotomy depends upon the woman. If the belly is large, so that the bandage fits snugly, it does good. If flat, where it can not be applied properly, it is of no use. He does not believe in the binder for obstetrical purposes. It should be sufficient merely to support, not compress. The latter action tends to jam the uterus in the pelvis, and displacements often, in consequence, result. A number of nurses have a reputation of preserving comeliness, but this is accomplished at the expense of involution, which is retarded as the resultant of congestion. The binder should be used only to make the patient comfortable; tighter than this does harm. He believes the support is useless in post partum hemorrhage. It is of importance not to keep the woman on the flat of her back, but tc let her turn from side to side.

Dr. Stuart McGuire, in closing the discussion, said he brought in the question of the obstetrical binder to give rise to discussion. Regarding the support after cœliotomy, the points to impress are thoroughness of suturing and length of time in bed. No matter how speedy the convalescence, the patient should be kept in bed from four to six weeks. The belt gives a false sense of security, should be used on fat women, and here for comfort.

MARK W. PEYSER, M. D.,
Secretary and Reporter.

